# Supplementary material for: Multimeric structure enables the acceleration of KaiB-KaiC complex formation induced by ADP/ATP exchange inhibition
Source: PLoS Comput Biol. 2022 Mar 7;18(3):e1009243. doi: 10.1371/journal.pcbi.1009243 (PMC8929707; doi:10.1371/journal.pcbi.1009243)
Supplement: Attachment — Submitted filename: ResponseR1.pdf [file pcbi.1009243.s001.pdf]

Dear Reviewers,

We are grateful to the reviewers for the time and effort dedicated to providing insightful comments on our manuscript. Below are our point-by-point answers to the reviewers' comments and concerns. In the response, we highlighted the reviewer’s comments in blue and italic font and the page/line numbers pointing the revised text in red. Please also note that, as a result of additional investigations, the title of our manuscript has been changed to "Multimeric structure enables the acceleration of KaiB-KaiC complex formation induced by the ADP/ATP exchange inhibition" and Abstract, Author summary, and Discussion have been thoroughly rewritten.

Sincerely yours,

Shin-ichi Koda
Shinji Saito

Response to Reviewer #1

Reviewer #1: In this manuscript, the authors develop a computational model to suggest that KaiC promotes KaiB binding by reducing exchange of ADP for ATP rather than actively promoting ATP hydrolysis to generate the ADP-bound state. Fundamental to their model is the assumption that cooperative binding of KaiB to the CI domain of KaiC happens only when all 6 CI domains are bound to ADP. Their model relies on five assumptions about KaiB-KaiC binding that are rooted in large part from prior structural or biochemical studies. However, while most of these assumptions have a strong foundation in experimental data, one of them, (i), is based on a crystal structure of the isolated CI hexamer and therefore lacks potential regulation by the C-terminal CII domain, calling into question the reliability of their assumption that CI domains can take on KaiB binding-competent or -incompetent conformations independently of one another. Overall, this manuscript presents some interesting concepts about the fundamental properties of KaiC that regulate cooperative binding of KaiB that are worth publishing if the caveats or concerns below can be addressed.

Major:
1. Support for their first (i) assumption on lines 129-131, that C1 domains can independently assume binding-competent or -incompetent conformations in the hexamer, is based on a crystal structure of isolated CI hexamers from ref. 22 (i.e., in the absence of CII domains). There is potentially a major flaw in the model, as it neglects to consider a possible role for the CII domain (and its phosphostate) in regulating CI domain conformation through ring-ring stacking or some other mechanism. The possibility of a confounding factor in their model from the CII domain should be clearly stated.

Response: We agree with this comment. The revised manuscript has noted that the crystal structures lack the C2 domain, and the full-length KaiC could behave differently (page 8, line 194-198). In addition, as a response to Reviewer #2's suggestion, we have extended the model to investigate another possible case where the C1 domain changes its conformation cooperatively in the full-length KaiC (page 8-10, line 194-233; page 14-17, line 343-393).

2. The observation in prior work (ref. 16) that the phosphomimetic KaiC-EE binds to KaiC with an apparent increased binding rate compared to WT KaiC also implies that CII phosphorylation state plays an important role in KaiB binding, which has been shown many times before. It's not clear how the authors come to the conclusion that the observation above suggests that inhibition of ADP/ATP exchange is the cause of acceleration in complex formation. Further support for this should be provided.

Response: Thanks to this comment, we recognized that the effect of the phosphorylation on C1 was not explained sufficiently. In the revised manuscript, we have devoted a new sub-subsection to provide a detailed explanation (page 11, line 248-264). Simply stated, the present model follows the phosphorylation effect employed in the model of Paijmans et al. (Ref. 23). As Paijmans et al. have discussed in detail, we assume in the manuscript that the ADP/ATP exchange of C1 is inhibited but the ATP hydrolysis is not changed in KaiC-EE because this assumption is consistent with the experimental result that KaiC-EE has a lowered ATPase activity (Ref. 26). If the ATP hydrolysis is significantly activated instead, the ATPase activity would be elevated in contrast to the experiment.

Minor:
1. The terminology used here can gets confusing (e.g., "inhibition of the backward process"). I would suggest using scientific terminology like ATP hydrolysis or product release to describe the forward and backward reactions instead.

Response: We thank the reviewer for this suggestion. In the revised manuscript, we have avoided the terms "forward" and "backward" as much as possible, unless the direction of the process should be explicitly stated.

2. On line 159, should it be "necessarily" instead of "necessary"?

Response: We thank the reviewer for letting us know the typo. We have deleted it during the revision.

Response to Reviewer #2

Reviewer #2: The authors address the question of how KaiB binding is coupled to the ATPase cycle in the KaiC CI domain using mathematical modeling. This is in an important question because it is at the heart of the negative feedback loop that allows sustained oscillation in the cyanobacterial clock. The study reaches a number of interesting conclusions, including an intriguing alternative interpretation for the biphasic binding observed when KaiB is added to KaiC in several already published experiments.

However, I feel that the central conclusion, that cooperativity in KaiB binding to a KaiC hexamer allows for inhibition of the ATP exchange reaction to result in faster binding kinetics to be based on assumptions about the system that are too restrictive. While the proposed scenario, where a cooperativity mechanism can lead to acceleration of the relaxation time of a system in scenarios where a non-cooperative elementary reaction could only be slowed is theoretically quite interesting, I do not see a good reason for ignoring the possibility that the specific mechanism in KaiC involves regulation of both the ATP hydrolysis reaction and the exchange reaction. That is, a phosphorylation state favorable to binding could both accelerate hydrolysis in CI and inhibit subsequent exchange of ADP for ATP.

Response: We appreciate the careful comment for our insufficient explanation of the phosphorylation effect on C1. As replied to Reviewer #1, we have devoted a new sub-subsection to provide a detailed explanation and stated that the present model follows the phosphorylation effect employed in Paijmans' model (Ref. 23) (page 11, line 248-264). As Paijmans et al. have discussed in detail, we assume in the manuscript that the ADP/ATP exchange of C1 is inhibited but the ATP hydrolysis is not changed in KaiC-EE because this assumption is consistent with the experimental result that KaiC-EE has a lowered ATPase activity (Ref. 26). If the ATP hydrolysis is significantly activated instead, the ATPase activity would be elevated in contrast to the experiment. In the real system, it is less likely that the ATP hydrolysis of C1 is not at all affected by C2. However, we ignore the phosphorylation dependence as a simplification in mathematical modeling, as noted in the revised manuscript (page 11, line 262-264).

In fact, existing data suggest that KaiC freshly loaded with ATP shows a burst of hydrolysis that might be consistent with such a scenario. The authors should revisit their analysis to consider this possibility. Is it a possible alternative explanation of the data?

Response: We thank the reviewer for casting this important question. We agree that there could be several ways to explain the burst of ATPase activity. However, we would like to confirm first that this phenomenon can be explained without changing the rate constant of ATP hydrolysis. Because the ADP production rate is given by the product of the rate constant and the amount of ATP bound to catalytic sites, the KaiC freshly loaded with ATP would show an increase of ATPase activity due to the latter factor, even if the rate constant is unchanged. In fact, Paijmans' model, which also adopts the invariant

ATP hydrolysis rate constant, can reproduce the initial burst of the ATPase activity (Fig 9B in Ref. 23. To be precise, this figure shows the amount of bound ATP in KaiC. However, the ADP production rate can be obtained by multiplying the invariant rate constant of ATP hydrolysis.). In conclusion, we consider that the phenomenon discussed here does not necessarily oblige the ATP hydrolysis rate constant of C1 to be changed on the phosphorylation of C2. Rather, to explain the reduction of ATPase activity in the steady state of KaiC-EE compared to KaiC-WT, it would be reasonable to assume that the ADP/ATP exchange is mainly affected by C2, as mentioned in the reply above.

> *Another point is that the scenario the authors proposed is quite extreme where stable binding of KaiB cannot occur unless it is at all 6 CI sites. i.e. a single catalytically dead subunit would completely prevent KaiB binding (is this plausible?). The authors should analyze whether the performance of the model degrades noticeably if the strict cooperativity is weakened.*

Response: We appreciate this insightful suggestion that led us to a deeper understanding of the present model. In the revised manuscript, we have extended the present model to have a variable threshold number of bound ADP required for the complex formation, fixed to six in the original manuscript (page 8-10, line 194-233). As a result, we have found that a broader class of the present model can reproduce the experimental data and have reached a more general conclusion about requirements for realizing the simultaneous promotion and acceleration of the complex formation (page 14-17, line 343-393). Moreover, we have proposed an experiment to determine the threshold number of bound ADP (page 18-19, line 431-458).

> *A minor remark: the authors comment that because mutations that change the rate of the CI ATPase catalytic cycle affect the period, the other processes involved in KaiB-KaiC interaction are fast. This seems too strong. There could be other processes involved in period determination that are on a similar timescale to the CI catalytic cycle.*

Response: We agree that the original manuscript was too assertive. In the revised manuscript, we have noted that the assumption is made for simplicity, and the processed assumed fast could have a comparable time scale to the ATPase activity of C1 in the real system (page 7, line 165-167).

Response to Reviewer #3

Reviewer #3: This manuscript by Koda and Saito presents a new mathematical model for part of the circadian clock in cyanobacteria. The cyanobacterial clock is fascinating since it can be reconstituted in vitro with KaiA, KaiB, and KaiC, as well as ATP. Many previous modeling papers have considered this system. However, this manuscript does seem to present a new part of the story in that it studies hydrolysis on the CI domain of KaiC. The CII domain has received much of the previous attention, yet we know that the CI domain and the binding of KaiB to sequester KaiA eventually is also an important step.

In general, the manuscript is well written. However, some work could be done in the results and methods sections to give placeholders for the readers who are not as familiar with the details of the cyanobacterial circadian clock.

Another benefit of the current manuscript is the use of Bayesian analysis. This is not new in cyanobacterial modeling (e.g., see the work of Rust, Dinner, and colleagues) but can indeed be a fundamental approach going forward.

Response: We thank the reviewer for the comment. We have added several references (Refs. 32-36), including the work of Rust, Dinner, and colleagues (Ref. 36), to show successful applications of Bayesian parameter estimation.

My primary concern is that they focus on a small part of the whole picture. It is left to the reader to see precisely how this gets interpreted into 24-hour oscillations. So, I think the readers would want to see the results integrated into a fuller model for a journal like PLoS Computational Biology. All the system details may not be needed, but this would allow us to understand how the developed model is consistent with other models and the overall systems biology of the system.

Response: We thank the reviewer for the constructive suggestion. However, in the revised manuscript, we have deferred the integration. Our results indicate that, in contrast to other models, the conformational transitions of C1 and C2 should be treated separately, and the interaction between them should be reconsidered in the integration. If these difficult problems are treated in the present study, they might obscure the focus. Instead, we have decided to deepen the understanding of the KaiB-KaiC complex formation by conducting a more comprehensive investigation, according to Reviewers #1 and #2's concerns. Furthermore, we have shown that Paijmans' model (Ref. 23), one of the successful models of the oscillator, is also compatible with the simultaneous promotion and acceleration of the complex formation as long as the parameters are appropriately modified (page 17-18, line 394-430). Although the parameters modified in the revised manuscript are arrhythmic and it is still unknown that Paijmans' model can achieve the oscillation and the complex formation focused here at the same time,

we believe that the present investigation would be a first step toward integrating the present model into a fuller model.

> *Additionally, I think the authors should consider how phosphorylation in the CII domain affects their model of the CI domain further. Even if these events occur at different times in the cycle, some mechanism needs to be proposed for how information is spread across the whole protein. The authors hint at this in future work, but I think it may be required here to convince readers of the importance of the modeling.*

Response: We appreciate the careful comment on the phosphorylation effect on C1. As replied to Reviewers #1 and #2, we have devoted a new sub-subsection to provide a detailed explanation and stated that the present model follows the phosphorylation effect employed in Paijmans' model (Ref. 23) (page 11, line 248-264). As Paijmans et al. have discussed in detail, we assume in the manuscript that the ADP/ATP exchange of C1 is inhibited but the ATP hydrolysis is not changed in KaiC-EE because this assumption is consistent with the experimental result that KaiC-EE has a lowered ATPase activity (Ref. 26). It would be ideal if we could propose a finer (i.e., atomic) mechanism regulating the ADP/ATP exchange of C1. However, we have not conducted any atomic investigation so far. Thus, in the present study, we have stated only the phosphorylation dependence of the rate constants, which can be inferred from experimental results.
