# Supplementary material for: Multimeric structure enables the acceleration of KaiB-KaiC complex formation induced by ADP/ATP exchange inhibition
Source: PLoS Comput Biol. 2022 Mar 7;18(3):e1009243. doi: 10.1371/journal.pcbi.1009243 (PMC8929707; doi:10.1371/journal.pcbi.1009243)
Supplement: Attachment — Submitted filename: ResponseR2.pdf [file pcbi.1009243.s002.pdf]

Dear Reviewers,

We are grateful to the reviewers for the valuable comments on our manuscript. Below are our point-by-point answers to the reviewers' comments and concerns. In the response, we highlighted the reviewer's comments in blue and italic font.

Sincerely yours,

Shin-ichi Koda
Shinji Saito

Response to Reviewer #1

1. The statement on line 56-58 should be supported with a citation of the KaiABC structures in refs. 14 and 15 (Snijder, J. et al. 2017, Science and Tseng, R. et al. 2017, Science).

Response: Following this comment, we added these two references.

2. The title might flow a bit better if 'the' was removed before "ADP/ATP exchange inhibition" or if it read "...induced by inhibition of ADP/ATP exchange."

Response: We thank the reviewer for correcting our English. Following this comment, we changed the title to "Multimeric structure enables the acceleration of KaiB-KaiC complex formation induced by ADP/ATP exchange inhibition."

Response to Reviewer #2

Reviewer #3: I think the authors have sufficient addresses the reviewer's comments. It would have been good to have a more in depth discussion of the various models of the whole Cyanobacterial clock rather than just to reference Paijman's model, especially as new simulations of the whole system were not added. Perhaps some of the other modeling could be discussed, for example the models now cited by the Rust group or the recently published model of Tyler et al. (Genome Biology)? At least a little more text about this could help.

Response: We appreciate this careful comment highlighting important previous models. In the revised manuscript, we discussed the model by Rust group by focusing on the interaction between the conformational states of C1 and C2 (page 22-23, line 551-567) and the model of Tyler *et al.* in a context of universal clock properties among species (page 24, line 594-601).
